# Detection of *Escherichia coli*, *Salmonella* species, and *Vibrio cholerae* in tap water and bottled drinking water in Isfahan, Iran

**DOI:** 10.1186/1471-2458-13-556

**Published:** 2013-06-07

**Authors:** Hassan Momtaz, Farhad Safarpoor Dehkordi, Ebrahim Rahimi, Amin Asgarifar

**Affiliations:** 1 Department of Microbiology, College of Veterinary Medicine, ShahreKord Branch, Islamic Azad University, P.O. Box: 166, ShahreKord, Iran; 2 Young Researchers Club, Islamic Azad University, ShahreKord Branch, ShahreKord, Iran; 3 Department of Food Hygiene, College of Veterinary Medicine, ShahreKord Branch, Islamic Azad University, ShahreKord, Iran; 4 Graduated of Veterinary Medicine, College of Veterinary Medicine, ShahreKord Branch, Islamic Azad University, ShahreKord, Iran

**Keywords:** *Escherichia coli*, *Salmonella* species, *Vibrio cholerae*, Water, PCR, Iran

## Abstract

**Background:**

The quality of drinking water has an important role in human infection and disease. This study was aimed at comparing polymerase chain reaction and culture in detecting *Escherichia coli*, *Salmonella* species and *Vibrio cholera* in tape water and bottled drinking water in various seasons in Isfahan province, Iran.

**Methods:**

A total of 448 water samples from tap water and bottled mineral water were taken over 6 months, from July 2010 to December 2010, and after filtration, samples were examined by culture and polymerase chain reaction methods for detection of *Escherichia coli*, *Salmonella* species*,* and *Vibrio cholerae*.

**Results:**

The culture method showed that 34 (7.58%), 4 (0.89%) and 3 (0.66%) of all 448 water samples were positive for *Escherichia coli*, *Salmonella* species*,* and *Vibrio cholera*, respectively. The *uidA* gene from *Escherichia coli*, *IpaB* gene from *Salmonella* species*,* and *epsM* gene from *Vibrio cholera* were detected in 38 (26.38%), 5 (3.47%), and 3 (2.08%) of 144 tap-water samples, respectively. *Escherichia coli* was detected in 8 (2.63%) of 304 samples of bottled drinking water from 5 companies. The water of southern part of Isfahan and company 5 had the highest prevalence of bacteria. The *Escherichia coli* water contamination was significantly higher (*P* < 0.05) in the hot seasons (July-August) than cold (November-December) seasons and in company 5 than other companies. There were significant differences (*P* < 0.05) for the prevalence of bacteria between the tap waters of southern part and tap waters of central part of Isfahan.

**Conclusions:**

This study showed that the polymerase chain reaction assays can be an extremely accurate, fast, safe, sensitive and specific approach to monitor drinking water quality from purification facilities and bottled water companies. Also, our study confirmed the presence of *Escherichia coli*, *Salmonella* species*,* and *Vibrio cholerae* as water-borne pathogens in tap water and bottled drinking water of Isfahan, Iran. The present study showed the important public health problem in Isfahan, Iran.

## Background

Despite modern techniques for disinfection, sanitation, and water purification, waterborne diseases still threaten human health. For all living organisms, water is the most vital and important matter for survival [1]. Today, in many under-developed and even developing countries, waterborne diseases still pose a major risk in drinking water. To the authors’ knowledge, the safety of commercial drinking water is generally dependent on the disinfection of natural water used at the factory. Sewage and pesticides can easily contaminate drinking water supplies. Therefore, monitoring the microbiological quality of drinking water is essential.

Studies showed that ingestion of water contaminated with coliforms such as *Escherichia coli* (*E. coli*), *Salmonella* species (*Salmonella* spp.), and *Vibrio cholerae* (*V. cholerae*) can create serious complications including diarrhea, enteritis, and even death, leading to high economic losses [2-6]. *Salmonella* spp., *V. cholerae,* and *E. coli* are classified as zoonotic agents. These bacteria are found almost everywhere. Humans, animals, and sewage can be sources of these bacteria. Contaminated water plays an important role in transmission of bacteria to humans. Using accurate, fast, safe, reliable, sensitive, and specific diagnostic methods in water purification facilities can have a decisive impact on ensuring the absence of microbial pathogens in tap water and bottled drinking water.

Among all diagnostic techniques such as culture, serology, and molecular methods, the last one is the fastest [7]. Molecular methods have high sensitivity [8], specificity [9] and safety [10]. Nonetheless, culture methods remain popular because of their ease and simplicity. Polymerase Chain Reaction (PCR) is one of the most widely used molecular methods for detection of a wide variety of microorganism in various clinical samples. The PCR assays have been developed for detection of *Salmonella* spp., *V. cholerae,* and *E. coli* in a wide variety of sample types such as water [4], food [11-13], milk [14,15], and stool [16-18].

The purposes of this study were to detect *Salmonella* spp., *V. cholerae,* and *E. coli* in tap water and bottled drinking water in Isfahan, Iran, and to introduce PCR assays as an approach for detection of these bacteria in water samples.

## Methods

### Sample collection

Isfahan province -with population of 4,800,000 and an area of 291,107,044 square kilometers - is the second big province of Iran and is located in central part of Iran among Iran’s central mountains and eastern hillside of the Zagros Mountains at the margin of Zayande-rood River.

The drinking water of this province is supplied from Zayande-rood River which is considered as surface water. It is probable that this water is contaminated with industrial and urban sewerage at the margin of this river. To our knowledge, except refinement (filtering and chlorine dissipate), there are no hygienic activities (for example radiation on drinking water) for control the quality of drinking water in Isfahan. Also, this river is the only watercourse of companies producing bottled drinking water in this province. Therefore, one of the sources of microbial contamination (such as *Escherichia coli*, *Salmonella* spp., and *Vibrio cholerae*) in this area is water. Besides, most of hospitals, especially at the central part of Isfahan, use from this water and it is important to free from any pathogens. In this study, total of 448 tap-water and bottled drinking water were examined over a period of 6 months, from July 2010 to December 2010. Totally, 144 tap-water samples were collected from four different geographical regions of Isfahan province. For each region, 36 samples were collected in 1000 mL glass bottles containing 0.5 g of sodium thiosulphate for dechlorination of the water. Also, 304 bottled drinking water were purchased from a five different companies, which use the same water system, the day that the experiment was conducted.

### Bacterial isolation and biochemical tests for the identification of E. coli, Salmonella spp., and V. cholera

For *Vibrio cholerae* isolation, two liters of water were filtered through 0.22 μm membranes, using 12 to 15 membranes per sample. Membranes were subsequently incubated in 100 mL of alkaline peptone water, pH 8.6, for 6-8 h at 35°C. Two loopfuls of broth were streaked on Thiosulfate Citrate Bile agar (TCBS agar, Difco) and incubated for 18 h at 37°C. Six to 12 typical colonies (yellow and 1 to 3 mm diameter) were transferred to nutritive soft agar (T1N1, 0.75% agar) and incubated for 24 h at 37°C. All colonies were stored at room temperature for further testing.

For *Salmonella* spp. Isolation, water was filtered through each Sterivex unit until the 0.22 μm pore-size membrane became occluded. Three filters were prepared at each site as follows. After the desired 1.5 L of water was filtered, water remaining inside the filter housing was forced out with a 50 mL syringe, and 2 mL of buffered peptone water BPW was added into the housing with a 20 mL syringe equipped with a 25 gauge, 5/8-in. [1.6-cm] needle. The entry and exit ports of the filter unit were capped, and the filter unit was incubated at 25°C for 4 h. After pre enrichment, 1 mL of the BPW was drawn off each filter and replaced with 1 mL of either 2 × Rappaport Vassilia (RV broth), 2 × Rappaport Vassilia Novobiocin (RVN) broth, or 2 × Dulcitol Selenite (DS) broth. After incubation at 43°C for 24 h, a loopful of each enrichment broth was streaked onto Xylose Lysine Decarboxylase- Novobiocin (XN) agar, Tryptic Soy Brilliant Green (TSBG) agar, and Tryptic Soy Brilliant Green- Sucrose (TSBG-S) agar. Plates were incubated at 43°C, and *salmonella*-like colonies were picked at 24 and 48 h and inoculated into Triple Sugar Iron (TSI) agar slants. The slants were incubated at 43°C for 24 h, and isolates which fermented only glucose were inoculated onto API 20E test strips (Analytab Products, Plainview, N.Y.) for identification to the *Salmonella* spp.

For *E. coli* isolation from water samples, each sample was labeled to show serial number, place of water, type of water as well as time and date of collection. Examination of the water samples was completed within 24 hours after collection using Standard Total Coliform Multiple-Tube (MPN) Fermentation Techniques. After determination of MPN the tubes showing growth were inoculated onto MacConkey, and EMB agar plates (Merck). After 24 h incubation at 35°C ± 0.5°C for 24 h ±2 h gram-negative microorganisms were isolated from MacConkey agar and EMB agar and determined at the species level using cytochrome oxidase, triple sugar iron agar, urea and indole tests as putatively *E. coli*.

### DNA extraction and PCR conditions

Purification of DNA directly from water samples filtered was achieved using a Genomic DNA purification kit (Fermentas, Germany) according to the manufacturer’s instructions.

Oligonucleotide primers specific for the *uidA* gene of *E. coli* encoding for beta-D-Glucuronidase (Forward: 5′-AAAACGGCAAGAAAAAGCAG-3′ and Reverse 5′-ACGCGTGGTTAACAGTCTTGCG-3′) [19], the *ipaB* gene of *Salmonella* spp. encoding the invasion plasmid antigen B (Forward : 5′-GGACTTTTTAAAAGCGGCGG-3′ and reverse: 5′-GCCTCTCCCAGAGCCGTCTGG-3′) [8] and the *epsM* gene of *V. cholerae* encoding the enterotoxin extracellular secretion protein of toxigenic *V. cholera* (Forward : 5′- GAATTATTGGCTCCTGTGCAGG-3′ and reverse: 5′-ATCGCTTGGCGCATCACTGCCC-3′) [8] were used, as they have been reported to be specific for the respective bacteria. For detection of *E. coli* O157:H7 serotype as major pathogen in *E. coli* isolates PCR assay was used based on the method which was described previously by Fode-Vaughan et al. [20]. Therefore, all of the positive *E. coli* strains were tested for presence of *E. coli* O157:H7 serotype.

The PCR reactions were performed in a total volume of 25 μL, including 1.5 mM MgCl2, 50 mM KCl, 10 mM Tris-HCl (pH 9.0), 0.1% Triton X-100, 200 μM dNTPs each (Fermentas), 25 pmoL of each *V. cholerae* specific primer or 50 pmoL of each of the *E. coli*- or *Salmonella* spp.-specific primers, 1.5 U of Taq DNA polymerase (Fermentas), and 3 μL (40-260 ng/μL) of DNA. Amplification reactions were carried out using a DNA thermo-cycler (Eppendorf Mastercycler 5330, Eppendorf-Nethel-Hinz GmbH, Hamburg, Germany) as following: for *V. cholerae* and *E. coli*: heat denaturation at 94°C for 2 min followed by 25 cycles of heat denaturation at 94°C for 1 min, primer annealing at 58°C for 1 min and DNA extension at 72°C for 1 min. After the last cycle, the samples were kept at 72°C for 2 min to complete the synthesis of all strands [4]. The PCR amplification for *Salmonella* spp. was performed as described by Kong et al. (2002): heat denaturation at 94°C for 2 min followed by 35 cycles of heat denaturation at 94°C for 1 min, primer annealing at 62°C for 1 min and DNA extension at 72°C for 2.5 min. This was followed by incubation at 72°C for 10 min and cooling at 4°C. Amplified samples were analyzed by electrophoresis (120 V/208 mA) in 1.5% agarose gel and stained by ethidium bromide. A molecular weight marker with 100 bp increments (100 bp ladder, Fermentas) was used as size standard.

### Sequencing

In order to confirm the PCR results, a sequencing method was used. For this reason, PCR products of some positive samples were purified with a High Pure PCR Product Purification Kit (Roche Applied Science, Penzberg, Germany) according to the manufacturer’s recommendations. Single DNA strands were sequenced with an ABI 3730 XL device and Sanger sequencing method (Macrogen, Seoul, South Korea). The sequence of each gene was aligned with the gene sequences recorded in the GenBank database on the NCBI.

### Statistical analysis

Statistical analysis was performed using SPSS/18.0 software for significant relationship between hot and cold seasons for occurrence of bacteria in water. Chi-square test was performed and differences were considered significant at values of *P* value < 0.05.

## Results and discussion

Due to the large area (291,107,044 square kilometers) and dry and desert climate of the province, samples were collected from four different regions of the province in various seasons, and from 5 drinking water companies.

The culture technique showed that 34 (23.61%), 4 (2.77%) and 3 (2.08%) out of 144 tap water and only 7 (2.3%) out of 304 bottled drinking water were positive for presence of *E. coli*, *Salmonella* spp.*,* and *V. cholerae*, respectively. The results of the PCR techniques showed that the specific *uidA*, *IpaB,* and *epsM* gene targets of *E. coli*, *Salmonella* spp.*,* and *V. cholera* were detected in 38 (26.38%), 5 (3.47%), and 3 (2.08%) of 144 tap water samples, respectively. (The positive samples for *V. cholerae* have been obtained by testing on river’s water). Only the *uidA* gene from *E. coli* was detected in 8 (2.63%) of 304 samples of bottled drinking water (Table 1). There were no O157:H7 serotype between the *E. coli* isolates.

**Table 1:** **Distribution of *Escherichia coli*, *Salmonella *spp., and *Vibrio cholerae *tap-water and bottled mineral water-positive samples from Isfahan province, Iran**

**Sample water**	**Area**	***E. coli***
		***Salmonella *spp.**
		V. cholerae*
	Western Isfahan (n = 36)	10
		2
		-
**Tap-water**	Central Isfahan (n = 36)	2
		1
		-
**(n = 144)**	Northern Isfahan (n = 36)	10
		-
		-
	Southern Isfahan (n = 36)	16
		2
		3
	Total (n = 144)	38 (26.38%)
		5 (3.47%)
		3 (2.08%)
**Bottled mineral water**	Company 1 (n = 56)	-
		-
		-
**(n = 304)**	Company 2 (n = 61)	3
		-
		-
	Company 3 (n = 46)	-
		-
		-
	Company 4 (n = 42)	-
		-
		-
	Company 5 (n = 69)	5
		-
		-
	Total (n = 304)	8 (2.63%)
		-
		-
**Total (n = 448)**	Total (n = 448)	46 (10.26%)
		5 (1.11%)
		3 (0.66%)

*Number of positive samples.

In the southern area of Isfahan, 44.44%, 5.55%, and 8.33% of tap water samples were positive for *E. coli*, *Salmonella* spp.*,* and *V. cholera*, respectively*.* Totally, 7.24% of the samples of company 5 were positive for *E. coli.* The southern area of Isfahan and company 5 had the highest incidence of water contamination. Our results showed that all of the 34 *E. coli*, 4 *Salmonella* spp., and 3 *V. cholera* isolates in culture methods, had also positive PCR results. All of the 7 *E. coli* isolates, had a positive PCR results. Besides, the PCR method detected *E. coli* and *Salmonella* spp.*,* in 4 and 1 samples more than the culture method in tap water. Also, the PCR technique detected *E. coli* in one bottled drinking water sample more than the culture method. Therefore, the PCR method had a higher accuracy for detection of these bacteria in water samples.

The 144 tap water and 304 bottled drinking water samples were collected in different seasons corresponding to July-August, September-October, and November-December. Among the tap water samples, 27 (18.75%), 14 (9.72%), and 5 (3.47%) were positive for these bacteria in the three seasons, respectively (Table 2).

**Table 2:** **Distribution of *Escherichia coli*, *Salmonella *spp., and *Vibrio cholerae *tap-water and bottled mineral water-positive samples from Isfahan province, Iran in different seasons**

**Sample water**	**Area**	July-August*	**September-October**	**November-December**	**Total positive**
	Western Isfahan (n = 36)	8	4	0	12 (33.33%)
**Tap-water**	Central Isfahan (n = 36)	2	1	0	3 (8.33%)
**(n = 144)**	Northern Isfahan (n = 36)	7	1	2	10 (27.77%)
	Southern Isfahan (n = 36)	10	8	3	21 58.33%)
	Total (n = 144)	27	14	5	46 (31.94%)
**Bottled mineral water**	Company 1 (n = 56)	0	0	0	0 (0.0%)
**(n = 304)**	Company 2 (n = 61)	2	1	0	3 (4.91%)
	Company 3 (n = 46)	0	0	0	0 (0.0%)
	Company 4 (n = 42)	0	0	0	0 (0.0%)
	Company 5 (n = 69)	2	2	1	5 (7.24%)
	Total (n = 304)	4	3	1	8 (2.63%)
**Total (n = 448)**	Total (n = 448)	31 (6.91%)	17 (3.79%)	6 (1.33%)	54 (12.05%)

^*^Number of positive samples.

Among the bottled drinking water samples, 4 (1.31%), 3 (0.98%), and 1 (0.32%) were positive in July-August, September-October, and November-December, respectively (Table 2). The tap water and bottled drinking water samples collected in July-August had the highest frequency of contaminating with these bacteria.

Statistical analysis showed a significant difference (*P* = 0.039) for the presence of bacterial contaminants between tap water from the southern part and those from central part of Isfahan. There was also a significant difference (*P* = 0.027) between the number of positive samples of bottled drinking water from company 5 and other companies. Besides, the number of contaminated tap water samples was significantly higher (*P* = 0.037) in July-August versus November-December. Totally, 46 (10.26%), 5 (1.11%), and 3 (0.66%) samples out of the 448 tap water and bottled drinking waters, were positive for *E. coli*, *Salmonella* spp.*,* and *V. cholerae,* respectively*.* Totally, 31 (6.91%), 17 (3.79%) and 6 (1.33%) of the samples were positive for the bacterial contamination in July-August, September-October, and November-December, respectively.

In summary, the present study showed that *E. coli*, *Salmonella* spp.*,* and *V. cholerae* are frequently isolated in water from the southern part of Isfahan province, and there were significant differences for the number of contaminated samples between the hot (July- August) the and cold (November-December) seasons (*P* < 0.05).

Our study showed that the PCR methods were more accurate, safe and rapid than culture methods. Analysis of data showed that the sensitivity and specificity of the PCR methods were higher than the culture assays (Tables 3, 4 and 5). Based on this table the sensitivity and specificity of the PCR method for detection of *E. coli*, *Salmonella* spp. or *V. cholera* in all 448 water samples were 100% and 99%, 100% and 99.7% and finally 100% and 100%, respectively. In addition to lower sensitivity and specificity of the culture method, this assay has the high risk for laboratories, low-speed, high time consuming and complexity of doing. Therefore, it is not recommended for isolation of *E. coli*, *Salmonella* spp., or *V. cholera* bacteria in drinking water.

**Table 3:** **Study the sensitivity and specificity of PCR method for detection of *E. coli***

	**Culture positive**	**Culture negative**	**Total**
PCR positive	34^*a)^	4 ^c)^	38
PCR Negative	0 ^b)^	410^**d)^	410
Total	34 ^a+b)^	414 ^c+d)^	448

^*^Sensitivity = $\frac{\left(a\right)}{\left(a+b\right)}$ = 100%.

^**^Specificity = $\frac{\left(d\right)}{\left(c+d\right)}$ = 99%.

**Table 4:** **Study the sensitivity and specificity of PCR method for detection of *Salmonella species***

	**Culture positive**	**Culture negative**	**Total**
PCR positive	4^*a)^	1 ^c)^	5
PCR Negative	0 ^b)^	443^**d)^	443
Total	4 ^a+b)^	444 ^c+d)^	448

^*^Sensitivity = $\frac{\left(a\right)}{\left(a+b\right)}$ = 100%.

^**^Specificity = $\frac{\left(d\right)}{\left(c+d\right)}$ = 99.7%.

**Table 5:** **Study the sensitivity and specificity of PCR method for detection of *V. cholerae***

	**Culture positive**	**Culture negative**	**Total**
PCR positive	^3*a)^	0^c)^	3
PCR Negative	0^b)^	^445**d)^	445
Total	3^a+b)^	445^c+d)^	448

^*^Sensitivity = $\frac{\left(a\right)}{\left(a+b\right)}$ = 100%.

^**^Specificity = $\frac{\left(d\right)}{\left(c+d\right)}$ = 100%.

The presence of *E. coli*, *Salmonella* spp., or *V. cholerae* in drinking water is a threat to human health. These bacteria can cause haemorrhagic colitis [21], diarrhea, nausea, abdominal cramps, fever, and vomiting [22], and cholera [23], respectively. The tap waters of Isfahan province are contaminated with these three pathogens. Previous study indicated that water borne diseases are responsible for about 20% of all deaths in children fewer than 5 years of age [24]. Several studies have been reported a statistically significant increase in gastrointestinal illness in populations that drink contaminated water with different types of coliform bacteria [25]. Therefore, checking the load of contaminants in water supplies and using the accurate techniques for disinfection is very important.

The Zayande-rood River is the main water source for some provinces of Iran including some areas of Chaharmahal Va Bakhtiari, Isfahan, Yazd, Ghom, and Kerman. This river comes from the Zagros Mountains. After passing through several towns, agricultural land, and industrial areas, reaches to the Isfahan steel company and then enters the Isfahan purification facility. Several sources of pollution exist along the path of the river. To our knowledge, the main sources of water pollution are probably the Isfahan steel company, towns, industrial factories and agricultural lands along the river. Although it seems unlikely, even pollution at the source of the river in the mountains can contaminates the drinking water in Isfahan province. Poor adherence to the principles of water purification and disinfection in the Isfahan water purification facility and the five mineral water companies may play important roles in contamination of drinking water. The water source, proper hygiene of workers at drinking water production facilities, and use of microbiological tests can contribute to reducing the microbial load of bottled drinking water.

The *E. coli*, *Salmonella* spp., and *V. cholerae* are found almost everywhere. Infected humans and animals, sewage, soil, and even aquaculture can be sources of water bacterial contamination. Several studies indicated that *E. coli*, *Salmonella* spp., and *V. cholerae* can infect many species of fish [26-28]. Death or disease due to enteric pathogens in fish populations can release these bacteria into the water. River water contains plentiful nutrients for bacterial growth. Plant material, fish and animal carcasses, garbage, and even urban sewage provide nutrients needed by bacteria for survival and growth.

To the best of our knowledge, the present study is the first report of detection of *E. coli*, *Salmonella* spp.*,* and *V. cholerae* in tap water and bottled drinking water in Iran. The observed prevalence of enteric bacteria in drinking water is one of the highest reported. Our results indicated a regional public health problem for those that use Zayande-rood River water and even some bottled drinking water. The results of this study showed that Isfahan’s drinking water is polluted, and serious steps are needed to solve the problem.

According to recommendations from the WHO (World Health Organization), CAWST (Center for Affordable Water and Sanitation Technology) and previous studies, drinking water must be free from *E. coli*, *Salmonella* spp., and *V. cholera*[29-31]. We recommend PCR assays as an accurate, safe, rapid, and trustworthy approach for microbial testing of drinking water.

The results of our study are generally in agreement with a recent report in South Africa [4], but they used samples from ground and surface waters while we reported a higher public health hazard. Momba et al. [4] reported that 75% and 25% of ground water samples were positive for *E. coli* and *V. cholerae,* respectively while only 25% of the surface water samples were positive for these bacterial strains. The results of the present study are in agreement with our previous investigation [5] which showed that the low microbial quality of drinking water in Isfahan province, Iran. Therefore, the occurrence of these bacteria in drinking water in Isfahan province is due to the fact that may be some of the water quality control systems need to be applied and performed in Iran.

In a recent study in New York, *Salmonella* spp. were detected in 12 (75%) of 16 study sites, and the authors noted that *Salmonella* spp. often are not detected in water samples by culture methods, even when they are present in significant numbers [32]. In Sudan, only 1 (0.25%) of 400 water samples was positive for *V. cholerae*[23], and in a study in the Netherlands, the *E. coli* was detected in 2 (7.4%) of 27 water samples collected between April to July 2004 [33]. The prevalence of bacterial contamination in these studies was lower than our investigation. Our results showed that 3 (0.66%) of 448 water samples were positive for *V. cholerae.* This bacterium was more frequent (66% of samples) in a study in Peru [34]. Studies showed that the salinity of water can affect the distribution of *V. cholerae*[34]. However, waters of the Zayande-rood River and the bottled drinking water in our study were fresh. Therefore, salinity does not play a significant role in presence of *V. cholerae*. Our results showed that water pollution in the warm months of the year is higher than the cold months. Similar seasonal patterns have been reported previously [35-37]. However, several studies had the difference seasonal pattern [38-40].

## Conclusion

We conclude with several general observations. First, if current conditions continued, water borne diseases will pose a serious public health problem in Isfahan province of Iran (especially the southern parts of Isfahan). Second, proper application of PCR techniques provides an accurate, safe, sensitive, specific, and fast diagnostic method that can be used to monitor the microbial load of drinking water. Third, constructing a database on the autochthonous microbial flora of each bottled drinking water source may allow quick identification of all autochthonous bacteria. Fourth, water pollution in Isfahan province of Iran has a seasonal pattern. Therefore, improving hygiene in warm seasons of the year is more essential. To our knowledge, using multiple filters on the sewage effluent pipes from factories, the Isfahan steel company, and aquaculture farms located along the Zayande-rood River may reduce the water pollution load. Finally, it can be concluded that the occurrence of *Salmonella* spp., *E. coli* and *V. cholerae* in tape-water and especially bottled drinking water in Isfahan province, Iran is due to the fact that may be some good manufacturing practices (GMPs), food safety and quality standards (good agricultural practices (GAPs), and the hazard analysis and critical control point (HACCP) system need to be applied and performed in most of Iranian factories or even refinery rooms to control growth of bacteria during harvesting, distribution and storage periods. The properly chlorinated of water should be monitored; storage and testing the microbial quality of drinking water should be modified according to what has been observed in the models. Therefore, many studies should have been performed on different Iranian drinking waters for study the presence of various water-borne pathogens.

## Competing interests

The authors declare that they have no competing interests.

## Authors’ contributions

The DNA extraction, PCR techniques and supporting of project were performed by HM, ER and FSD and Samples collection, culture, Statistical analysis and writing of manuscript were performed by FSD and AA. All authors read and approved the final manuscript.

## Pre-publication history

The pre-publication history for this paper can be accessed here:

http://www.biomedcentral.com/1471-2458/13/556/prepub

## References

[B1] Duset AG da Silva MP Zietsman I Coping with hygiene in South Africa, a water scarce country Int J Environ Health Res 2003 13 S95 S105 10.1080/0960312031000102859 12775385

[B2] Cherry WB Hanks JB Thomason BM Murlin AM Biddle JW Croom JM *Salmonella* as an index of pollution of surface waters Appl Microbiol 1872 24 334 340 4562473 10.1128/am.24.3.334-340.1972 PMC376520

[B3] Hunter P Water-borne disease: epidemiology and ecology 1997 Chichester: Wiley 384

[B4] Momba MN Malakate VK Theron J Abundance of pathogenic *Escherichia coli, Salmonella typhimurium* and *Vibrio cholerae* in Nkonkobe drinking water sources J Water Health 2006 4 289 296 17036837 10.2166/wh.2006.011

[B5] Momtaz H Rahimi E Moshkelani S Detection of *Pseudomonas aeruginosa* by PCR in tap-water and bottled mineral water in the Isfahan province of Iran Water Sci Thechnol Water Supply 2011 11 642 646 10.2166/ws.2011.108

[B6] Pegram GC Rollins N Espey Q Estimating the cost of diarrhoea and epidemic dysentery in KwaZulu-Natal and South Africa Water South Africa 1998 24 11 20

[B7] Al Dahouk S Tomaso H Nöckler K Neubauer H The detection of *Brucella* spp. using PCR-ELISA and real-time PCR assays Clin Lab 2001 50 387 394 15330505

[B8] Kong RY Lee SK Law TW Law SH Wu RS Rapid detection of six types of bacterial pathogens in marine waters by multiplex PCR Water Res 2002 36 2802 2812 10.1016/S0043-1354(01)00503-6 12146868

[B9] Bomfim MR Barbosa-Stancioli EF Koury MC Detection of pathogenic leptospires in urine from naturally infected cattle by nested PCR Vet J 2008 178 251 256 10.1016/j.tvjl.2007.07.029 17869555

[B10] Theron J Cilliers J Du Preez M Brözel VS Venter SN Detection of toxigenic *Vibrio cholerae* from environmental water samples by an enrichment broth cultivation-pit-stop semi-nested PCR procedure J Appl Microbiol 2000 89 539 546 10.1046/j.1365-2672.2000.01140.x 11021588

[B11] Ferretti R Mannazzu I Cocolin L Comi G Clementi F Twelve-hour PCR-based method for detection of *Salmonella* spp. in food Appl Environ Microbiol 2001 67 977 978 10.1128/AEM.67.2.977-978.2001 11157272 PMC92676

[B12] Fratamico PM DebRoy C Miyamoto T Liu Y PCR detection of enterohemorrhagic *Escherichia coli* O145 in food by targeting genes in the *E. coli* O145 O-antigen gene cluster and the shiga toxin 1 and shiga toxin 2 genes Foodborne Path Dis 2009 6 605 611 10.1089/fpd.2008.0254 19435408

[B13] Lotfy NM Hassanein MA Abdel-Gawad FKH El-Taweel GE Bassem SM Detection of *Salmonella* Spp in aquatic insects, fish and water by MPN-PCR World J Fish Marine Sci 2011 3 58 66

[B14] Daly P Collier T Doyle S PCR-ELISA detection of *Escherichia coli* in milk Lett Appl Microbiol 2002 34 222 226 10.1046/j.1472-765x.2002.01074.x 11874546

[B15] Perelle S Dilasser F Malorny B Grout J Hoorfar J Fach P Comparison of PCR-ELISA and LightCycler real-time PCR assays for detecting Salmonella spp. in milk and meat samples Mol Cell Probes 2004 18 409 420 10.1016/j.mcp.2004.07.001 15488381

[B16] Albert MJ Islam D Nahar S Qadri F Falklind S Weintraub A Rapid detection of *Vibrio cholerae* O139 Bengal from stool specimens by PCR J Clin Microbiol 1997 35 1633 1635 9163504 10.1128/jcm.35.6.1633-1635.1997 PMC229809

[B17] Chiu CH Ou JT Rapid identification of *Salmonella* serovars in feces by specific detection of virulence genes, *invA* and *spvC*, by an enrichment broth culture-multiplex PCR combination assay J Clin Microbiol 1996 34 2619 2622 8880536 10.1128/jcm.34.10.2619-2622.1996 PMC229337

[B18] Ramotar K Waldhart B Church D Szumski R Louie TJ Direct detection of verotoxin-producing *Escherichia coli* in stool samples by PCR J Clin Microbiol 1995 33 519 524 7751350 10.1128/jcm.33.3.519-524.1995 PMC227983

[B19] Tsai YL Palmer CJ Sangermano LR Detection of *Escherichia coli* in sewage and sludge by polymerase chain reaction Appl Environ Microbiol 1993 59 353 357 8434906 10.1128/aem.59.2.353-357.1993 PMC202112

[B20] Fode-Vaughan KA Maki JS Benson JA Collins ML Direct PCR detection of *Escherichia coli* O157:H7 Lett Appl Microbiol 2003 37 239 243 10.1046/j.1472-765X.2003.01386.x 12904226

[B21] Kerr M Fitzgerald M Sheridan JJ McDowell DA Blair IS Survival of *Escherichia coli* O157:H7 in bottled natural mineral water J App Microbiol 1999 87 833 841 10.1046/j.1365-2672.1999.00928.x 10664908

[B22] Ocepek M Pate M Kušar D Hubad B Avberšek J Logar K Lapanje A Zrimec A Comparison of DNA extraction methods to detect *Salmonella* spp. in tap water Slov Vet Res 2011 48 93 98

[B23] Shanan S Abd H Hedenström I Saeed A Sandström G Detection of *Vibrio cholerae* and *Acanthamoeba* species from same natural water samples collected from different cholera endemic areas in Sudan BMC Res Notes 2011 4 109 10.1186/1756-0500-4-109 21470437 PMC3080310

[B24] Bourne DE Coetzee N An atlas of potentially water related diseases in South Africa 1990 Pretoria: Water Research Commission

[B25] Payment P Siemiatycki J Richardson L Renaud G Franco E Prevost M A prospective epidemiological study of gastrointestinal health effects due to the consumption of drinking water Int J Environ Health Res 1997 7 5 31 10.1080/09603129773977

[B26] Amy PS Hiatt HD Survival and detection of bacteria in an aquatic environment Appl Environ Microbiol 1989 55 788 793 2658805 10.1128/aem.55.4.788-793.1989 PMC184203

[B27] Faruque SM Albert MJ Mekalanos JJ Epidemiology, genetics, and ecology of toxigenic *Vibrio cholerae* Microbiol Mol Biol Rev 1998 62 1301 1314 9841673 10.1128/mmbr.62.4.1301-1314.1998 PMC98947

[B28] Martinelli Filho JE Lopes RM Rivera ING Colwell RR *Vibrio cholerae* O1 detection in estuarine and coastal zooplankton J Plankton Res 2011 33 51 62 10.1093/plankt/fbq093

[B29] World Health Organization (WHO) Guidelines for drinking-water quality 2011 Fourth Switzerland: WHO Library Cataloguing-in-Publication Data

[B30] CAWST Center for affordable water and sanitation technology 2009 Canada: Introduction to drinking water quality testing a cawst training manual

[B31] Ashbolt NJ Grabow WOK Snozzi M Fewtrell L, Bartram J Indicators of microbial water quality Water quality: guidelines, standards and health – assessment of risk and risk management for water-related infectious disease 2001 WHO Water Series. London: IWA Publishing 289 315

[B32] Knight IT Shults S Kaspar CW Colwell RR Direct detection of *salmonella* spp. In estuaries by using a DNA probe Appl Environ Microbiol 1990 56 1059 1066 2339868 10.1128/aem.56.4.1059-1066.1990 PMC184343

[B33] Heijnen L Medema G Quantitative detection of *E. coli, E. coli* O157 and other shiga toxin producing *E. coli* in water samples using a culture method combined with real-time PCR J Water Health 2006 4 487 498 17176819

[B34] Lipp EK Rivera IN Gil AI Espeland EM Choopun N Louis VR Russek-Cohen E Huq A Colwell RR Direct detection of *Vibrio cholerae* and *ctxA* in Peruvian coastal water and plankton by PCR Appl Environ Microbiol 2003 69 3676 3680 10.1128/AEM.69.6.3676-3680.2003 12788781 PMC161524

[B35] Anderson IC Rhodes MW Kator HI Seasonal variation in survival of *Escherichia coli* exposed in situ in membrane diffusion chambers containing filtered and nonfiltered estuarine water Appl Environ Microbiol 1983 45 1877 1883 6349528 10.1128/aem.45.6.1877-1883.1983 PMC242553

[B36] Heidelberg JF Heidelberg KB Colwell RR Seasonality of Chesapeake Bay bacterioplankton species Appl Environ Microbiol 2002 68 5488 5497 10.1128/AEM.68.11.5488-5497.2002 12406742 PMC129892

[B37] Suwandana E Kawamura K Tanaka K Sakuno Y Raharjo P *Escherichia coli* and biophysicochemical relationships of seawater and water pollution index in the Jakarta Bay Am J Environ Sci 2011 7 183 194 10.3844/ajessp.2011.183.194

[B38] Abakpa GO Umoh VJ Ameh JB Prevalence OF *Salmonella* spp in some environmental samples from some households engaged in livestock farming in some parts of Zaria, Nigeria Continental J Microbiol 2011 5 6 11

[B39] Ahmed W Neller R Katouli M Population similarity of *enterococci* and *Escherichia coil* in surface waters: a predictive tool to trace the sources of fecal contamination J Water Health 2006 4 347 356 17036842 10.2166/wh.2006.042

[B40] Wilkes G Edge TA Gannon VP Jokinen C Lyautey E Neumann NF Ruecker N Scott A Sunohara M Topp E Lapen DR Associations among pathogenic bacteria, parasites, and environmental and land use factors in multiple mixed-use watersheds Water Res 2011 45 5807 5825 10.1016/j.watres.2011.06.021 21889781

